# Novel Homozygous *ADAMTS2* Variants and Associated Disease Phenotypes in Dogs with Dermatosparactic Ehlers–Danlos Syndrome

**DOI:** 10.3390/genes13112158

**Published:** 2022-11-19

**Authors:** Jared A. Jaffey, Garrett Bullock, Juyuan Guo, Tendai Mhlanga-Mutangadura, Dennis P. O’Brien, Joan R. Coates, Rochelle Morrissey, Robert Hutchison, Kevin S. Donnelly, Leah A. Cohn, Martin L. Katz, Gary S. Johnson

**Affiliations:** 1 Department of Specialty Medicine, College of Veterinary Medicine, Midwestern University, Glendale, AZ 85308, USA; 2 Department of Veterinary Pathobiology, College of Veterinary Medicine, University of Missouri, Columbia, MO 65211, USA; 3 Department of Veterinary Medicine and Surgery, College of Veterinary Medicine, University of Missouri, Columbia, MO 65211, USA; 4 East Bay SPCA, 8323 Baldwin St., Oakland, CA 94621, USA; 5 Animal Clinic Northview, 36400 Center Ridge Rd., North Ridgeville, OH 44039, USA; 6 Neurodegenerative Diseases Research Laboratory, Department of Ophthalmology, University of Missouri, Columbia, MO 65212, USA

**Keywords:** collagen fibril, skin, tendon, ligament, cornea

## Abstract

Tissue fragility, skin hyperextensibility and joint hypermobility are defining characteristics of Ehlers–Danlos syndrome (EDS). Human EDS is subclassified into fourteen types including dermatosparactic EDS, characterized by extreme skin fragility and caused by biallelic *ADAMTS2* mutations. We report two novel, *ADAMTS2* variants in DNA from EDS-affected dogs. Separate whole-genome sequences from a Pit Bull Terrier and an Alapaha Blue Blood Bulldog each contained a rare, homozygous variant (11:2280117delC, CanFam3.1), predicted to produce a frameshift in the transcript from the first coding *ADAMTS2* exon (c.10delC) and a severely truncated protein product, p.(Pro4ArgfsTer175). The clinical features of these dogs and 4 others with the same homozygous deletion included multifocal wounds, atrophic scars, joint hypermobility, narrowed palpebral fissures, skin hyperextensibility, and joint-associated swellings. Due to severe skin fragility, the owners of all 6 dogs elected euthanasia before the dogs reached 13 weeks of age. Cross sections of collagen fibrils in post-mortem dermal tissues from 2 of these dogs showed hieroglyphic-like figures similar to those from cases of severe dermatosparaxis in other species. The whole-genome sequence from an adult Catahoula Leopard Dog contained a homozygous *ADAMTS2* missense mutation, [11:2491238G>A; p.(Arg966His)]. This dog exhibited multifocal wounds, atrophic scars, and joint hypermobility, but has survived for at least 9 years. This report expands the spectrum of clinical features of the canine dermatosparactic subtype of EDS and illustrates the potential utility of subclassifying canine EDS by the identity of gene harboring the causal variant.

## 1. Introduction

The Ehlers–Danlos syndromes (EDS) are a group of connective tissue disorders characterized by tissue fragility, skin hyperextensibility, and joint hypermobility [1]. There is extensive phenotypic and genetic heterogeneity in humans with EDS [2]. The current system for distinguishing the various forms of human EDS recognizes 14 subtypes based on differences in medical histories, laboratory findings, patterns of inheritance, and the identities of the genes that harbor the causal variants [2]. Assignment of the correct EDS subtype is important because it helps establish the patient’s prognosis and the most appropriate medical management [3]. Because various clinical signs and ultrastructural features are shared among the different EDS subtypes, medical histories and results from laboratory procedures may be insufficient to establish a definitive diagnosis [2,4]. Therefore, the identification of the molecular-genetic cause is recommended whenever feasible [3,4]. At least 21 different genes have been reported to harbor variants that cause human EDS [2,5,6].

The clinical and genetic heterogeneity in canine EDS is likely to be as extensive as it is in the human disease complex. Although canine EDS has been described in numerous publications [7,8,9,10,11,12,13,14,15,16,17,18,19,20,21,22,23,24,25,26,27,28,29,30,31,32], the accumulated knowledge has been too sparse and fragmented to support the classification of EDS into distinct subtypes in dogs. Thus, it is not currently possible to determine which information from previously published reports in dogs is most applicable to new cases. Recent advances in the generation and interpretation of DNA sequence data have made it feasible to distinguish subtypes of canine EDS based on the identity of the genes that harbor the causative alleles [4]. To date, six genetic variants have been reported to be likely causes of canine EDS. Two different *COL5A1* variants and a *COL5A2* variant were found in the heterozygous state in independent canine EDS cases, suggesting that these cases correspond to the “Classical” subtype of human EDS [2,7,9]. Another case of canine EDS had compound heterozygous *TNXB* variants, suggesting a correspondence to the “Classical-like” human subtype [2,8]. A homozygous *ADAMTS2* variant occurred in another canine EDS case, indicating correspondence to the “Dermatosparaxis” human EDS subtype [2,24].

Dermatosparactic EDS is caused by biallelic *ADAMTS2* variants that result in a deficiency of procollagen-I-N-proteinase, an enzyme that excises the N-terminal propeptide from the α-chains of fibrillar collagens [33,34]. Removal of the N-terminal propeptide is necessary for proper assembly of highly organized, fully functional collagen fibrils that provide tensile strength to connective tissue in skin and other organs [35]. While a wide spectrum of clinical signs has been associated with dermatosparaxis, the most common clinical manifestations of this subtype include hyperelasticity and extreme fragility of the skin resulting in large lacerations from minor trauma [34,36]. A 1982 report showed ultrastructural changes characteristic of dermatosparactic EDS in cross sections of collagen fibrils from the dermis of a dog; however, no clinical information was provided [23]. A more recent report described the clinical history of a Doberman Pinscher puppy with EDS likely caused by a homozygous *ADAMTS2* nonsense mutation [24]. Here, we expand the knowledge about the canine dermatosparaxis subtype of EDS by reporting two novel, likely-causal *ADAMTS2* variants and describing the disease phenotypes for seven dogs carrying these variants in the homozygous state.

## 2. Materials and Methods

### 2.1. Animal Population, Criteria for Selection, and Sample Acquisition

With the goal of expanding our understanding of the phenotypic diversity and related molecular-genetic causes of EDS in dogs, we have collected clinical records, tissues (biopsy and necropsy), and blood samples as a source of DNA from affected dogs since 2017. Dogs were eligible for inclusion if they had a tentative diagnosis of EDS based on at least two of the following three clinical signs: skin hyperextensibility, joint hypermobility, and skin fragility as indicated by a history of lacerations from minor trauma. Samples for DNA analysis were also collected from unaffected dogs with known familial relationships to dogs with EDS. Thus far, we have generated and analyzed whole genome sequences with DNA from 26 dogs that exhibited signs of EDS. The current study includes three of these dogs and four of their EDS-affected relatives. Experiments were performed in accordance with the relevant guidelines and regulations set forth by the University of Missouri Institutional Animal Care and Use Committee represented by the approved study protocol (#10125) and signed owner consent.

EDTA-anticoagulated venous blood samples or buccal swabs on FTA Elute cards (Whatman) were sent to the University of Missouri for molecular-genetic studies. Two dogs were donated by their owner for examination and euthanasia because of severe skin fragility. Tissue samples from a 10.5-month-old Dachshund, euthanized for reasons unrelated to the present study, were used as normal controls for histopathologic and ultrastructural studies. Archived DNA samples from 492 Alapaha Blue Blood Bulldogs and 217 Pit Bull Terriers were genotyped for the candidate variant: 11:2280117delC (CanFam3.1).

### 2.2. Molecular Genetic Analysis

Previously described methods were used to isolate DNA samples from EDTA anti-coagulated blood or from FTA Elute cards [37,38]. To generate whole genome sequences, individual DNA samples from three affected dogs (Dogs 1, 3, and 7) were submitted to the University of Missouri Genomics Technology Core Facility for the preparation of Illumina TruSeq PCR-free paired-end libraries with ~400 bp inserts. Dogs 1 and 3 were sequenced on an Illumina NextSeq500 platform producing 188 million and 204 million reads, respectively. Dog 7 was sequenced on an Illumina NovaSeq 6000 platform with producing 411 million reads.

The Burrows-Wheeler Aligner (BWA-mem) was used to map the sequence reads to a canine reference genome assembly (CanFam3.1) [39]. The reads were then sorted with SAMtools (ver. 1.11), and PCR duplicates were marked with Picard tools (ver. 2.23.8). A modified Genome Analysis Tool Kit (GATK ver. 3.8) best practices pipeline was used for realignment, recalibration, and variant calling. To help identify rare variants in the affected sample sequences, an additional 4114 canine whole genome sequences were obtained from the NCBI Sequence Read Archive (SRA) (https://trace.ncbi.nlm.nih.gov/Traces/sra/sra.cgi, accessed on 15 March 2022) and used as controls. The SRA accession numbers for all 4117 whole genome sequences used in this analysis are provided in the Supplemental Table S1. Variants in each of the control samples were called individually with GATK HaplotypeCaller in the gVCF mode. All sample gVCF files were joined with GATK CombineGVCFs, and jointly genotyped using GATK GenotypeGVCFs. Functional effects of the called variants were predicted with SnpEff software together with Ensembl annotation. SnpSift software was used for filtering low quality variants and extracting annotated variants for the affected samples. Variant reports were generated by tabulating the annotated output to a Microsoft Excel spreadsheet with GATK VariantsToTable. Candidate variants were visually inspected with the Integrative Genomics Viewer (IGV, ver. 2.8.10).

A 2-step allelic discrimination assay [40] was used to genotype DNA samples from individual dogs for the *ADAMTS2* variant identified in Alapaha Blue Blood Bulldogs and Pit Bull Terriers. The first step was PCR amplification with primers *5′-AGCTGCGGTTTGGCTCCAGCT-3′* and *5′-CTGCCGCGGGGCTTTC-3′*. These amplifications were conducted in 50 µL volumes with an AccuPrime GC-Rich DNA Polymerase Kit (Invitrogen) and included an initial denaturation at 95 °C for 3 min, followed by 35 cycles of denaturation at 95 °C for 30 s, primer annealing at 60 °C for 30 s, extension at 72 °C for 1 min, and a final extension at 72 °C for 10 min. Next, 8 µL of amplicons from the first stage amplification were used as the template for the second stage of the assay, which was conducted in 25 µL volumes with a custom-designed TaqMan SNP Genotyping Assay reagent on a StepOnePlus Real-Time PCR System (Applied Biosystems). Reaction mixtures consisted of the amplified DNA template, (36 µM) primers, and (8 µM) probes in TaqMan Genotyping Master Mix. The PCR primer sequences were *5′-CGGTCCGGCTGCCAT-3′* and *5′-GCAGCAGCAGCAGCAG-3′*. The competing probes were *5′-VIC-CCGCCGGCGGATC-NFQ-3′* (reference allele) and *5′-FAM-CCCGCCGCGGATC-NFQ-3′* (mutant allele).

### 2.3. Post-Mortem Sample Processing and Analysis

Two donated 8-week-old littermate Alapaha Blue Blood Bulldog puppies (Dogs 5 and 6) were examined and euthanized on the day of arrival at the University of Missouri. Humane euthanasia was performed with intravenous administration of pentobarbital. Tissues were collected immediately after euthanasia, placed in a fixative containing 2% glutaraldehyde, 1.12% paraformaldehyde, 130 mM sodium cacodylate, 1 mM CaCl_2_, pH 7.4, and incubated at room temperature for at least 48 h. A small slice of each sample, approximately 3 mm × 3 mm × 1 mm, was then washed in 170 mM sodium cacodylate, pH 7.4, post-fixed with osmium tetroxide, and embedded in epoxy resin.

Sections of the embedded samples were cut at a thickness of 0.25 µm, mounted on glass slides, stained with toluidine blue, and examined with light microscopy to identify areas of interest. Light microscopy images of these sections were obtained using a Leica DMI 6000B microscope. The tissue blocks were then trimmed to leave only the areas of interest on the block face. Sections of the trimmed blocks were then cut at thicknesses of 70 to 90 nm and mounted on copper electron microscopy grids. These sections were stained with uranyl acetate and lead citrate and were then examined with a JEOL JEM-1400 transmission electron microscope equipped with a Gatan digital camera.

## 3. Results

### 3.1. Clinical Histories and Postmortem Findings

#### 3.1.1. Dogs 1 and 2

Two 5-day-old male dogs along with their unaffected mother and five littermates were relinquished to a regional animal shelter. Shelter staff members considered the dogs to be Pit Bull Terriers, based on the appearance of the mother. There were no abnormalities identified in the mother or the seven puppies by physical examination at the time of admission. Initially, all the puppies were housed with the mother. Wounds on the head and neck of Dogs 1 and 2 were first identified at 2 weeks of age. These wounds were seen by staff members to occur while the puppies played with littermates or when the mother would pick them up by the neck to move them. The five other puppies did not develop any wounds. Dogs 1 and 2 were then housed in separate foster homes but new wounds continued to develop with each puppy. The wounds healed normally by second intention leading to the accumulation of atrophic scars. Both puppies were subsequently euthanized at 8 weeks old because of their severe skin fragility. At the time of euthanasia, both puppies had an ideal body condition, as well as normal temperature, pulse, and respiratory rates. Thoracic auscultation and abdominal palpation were unremarkable. Subjectively, Dog 1 seemed to be more severely affected than Dog 2. Dog 1 had skin hyperextensibility, numerous wounds at various stages of healing, atrophic scars, joint hypermobility, a large hematoma on the pinna of the right ear, narrowed palpebral fissures, swelling of both tarsi and all four paws. The joints did not seem overtly painful, but Dog 1 demonstrated difficulty walking and was intermittently ataxic. Dog 2 had skin hyperextensibility, numerous wounds at various stages of healing, atrophic scars, and a large, ruptured hematoma on the right ear pinna. Notably, Dog 2 did not have joint swelling or difficulty walking; however, a full orthopedic examination was not performed. Four of the five unaffected littermates were alive at 18 months of age. One littermate was euthanized because of behavioral issues but did not demonstrate any clinical signs associated with EDS.

#### 3.1.2. Dogs 3 and 4

Two female Alapaha Blue Blood Bulldog littermates were part of a litter of four siblings. Dog 3 was presented to the primary care veterinarian at 4 weeks old for evaluation of swellings around both elbows and numerous small skin wounds located on the elbows, face, and ear pinna. This dog was diagnosed with suspected cellulitis and treated with amoxicillin/clavulanic acid. To avoid additional wounds, Dog 3 was then separated from the littermates. Nonetheless, the puppy continued to develop wounds from minimal trauma (e.g., bumping into a wall or being picked up by the owner). These wounds healed normally by second intention, forming atrophic scars. The puppy was evaluated again at 8 weeks old and there were painful swellings around both elbows and tarsi. The puppy continued to be active but had a noticeable lameness in all four limbs.

The clinical history of the littermate (Dog 4) was reported by the owner, as this puppy was not evaluated by the primary care veterinarian. The clinical signs in Dog 4 were subjectively less severe and had a delayed onset compared to the sibling (Dog 3). The wounds first became apparent at 5 weeks old. This puppy was then separated from the unaffected littermates and housed with Dog 3. Both puppies continued to accumulate wounds along the face, ear pinna, neck, abdomen, dorsum, and dorsal aspect of the paws. At 10 weeks old, non-painful swellings around both tarsi were identified.

**Figure 1:**
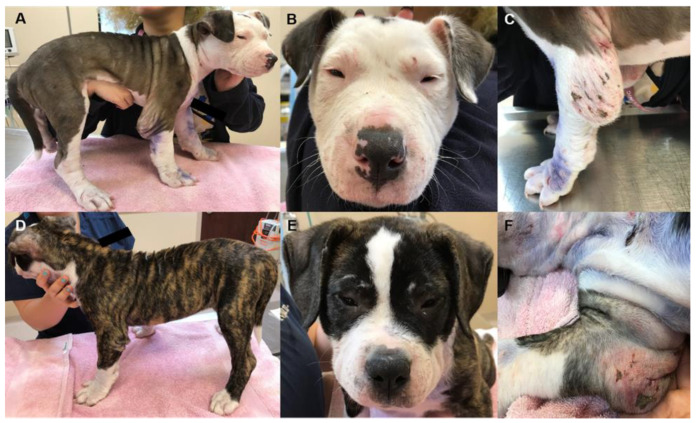
Representative images from two female, 12-week-old, Alapaha Blue Blood Bulldog littermates (Dog 3 (**A**–**C**); Dog 4 (**D**–**F**)) illustrating multifocal wounds at varying stages of healing, atrophic scars, narrowed palpebral fissures, and joint swellings.

When 12 weeks old, Dogs 3 and 4 were presented to their primary care veterinarian for humane euthanasia requested by the owner primarily because of severe skin fragility. On physical examination, both puppies were mentally appropriate, had an ideal body condition, as well as normal temperature, pulse, and respiratory rates. The palpebral fissures for both dogs were nearly closed (Figure 1). Thoracic auscultation and abdominal palpation were unremarkable. The oral cavity and dentition were age-appropriate in both puppies. Both puppies had large, painful, fluctuant swellings on the caudal aspect of both elbows and tarsi (Figure 1). These swellings appeared red, bruised, and had multiple small wounds at various stages of healing. Dog 4 also had swelling of all four paws. Both puppies had numerous wounds and atrophic scars located along the caudal aspects of both elbows and tarsi, face, ear pinnae, abdomen, dorsum, and the dorsal aspect of all paws. The skin was hyperextensible, especially along the dorsum and caudal aspects of the elbows. Joint hypermobility was identified in the carpi and tarsi of both dogs. Dog 3 was subjectively lame on all limbs but was more severe in the forelimbs. Dog 4 was not lame but did appear to be ataxic, which coincides with the owner report that the puppy was “wobbly.” The remainder of the physical examination was unremarkable.

#### 3.1.3. Dogs 5 and 6

Two 8-week-old littermate Alapaha Blue Blood Bulldogs were donated by their owner to the University of Missouri, College of Veterinary Medicine for examination and euthanasia because of severe skin fragility. Detailed medical histories before the time of donation were not provided. The puppies were examined and euthanized on the same day they arrived. On examination, both the male (Dog 5) and the female puppy (Dog 6) were mentally appropriate, had an ideal body condition, and normal temperature, pulse, and respiratory rates. Thoracic auscultation and abdominal palpation were unremarkable.

**Figure 2:**
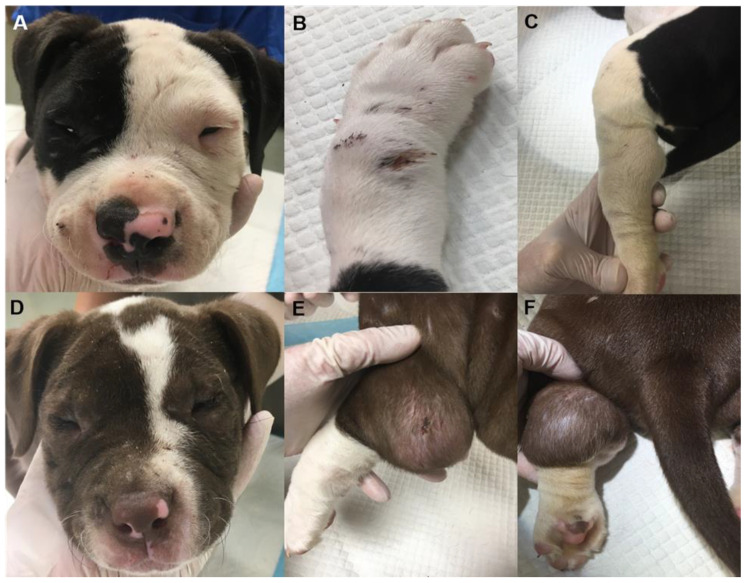
Representative images from two, 8-week-old, Alapaha Blue Blood Bulldog littermates (Dog 5 (**A**–**C**); Dog 6 (**D**–**F**)) illustrating multifocal wounds at varying stages of healing, atrophic scars, narrowed palpebral fissures, joint swellings and hypermobility.

Both dogs had an ophthalmic examination performed by a board-certified veterinary ophthalmologist (K.S.D.). Dog 5 demonstrated a positive direct and indirect pupillary light reflex in both eyes. A cotton ball was successfully tracked when thrown into the puppy’s visual field, indicating the puppy was visual. A Schirmer tear test II was performed resulting in 21 and 20 mm/min of wetting in the right eye and left eye, respectively. Intraocular pressures of 15 mmHg in the right eye and 17 mmHg in the left eye were measured with an iCare Tonovet rebound tonometer. Fluorescein staining was negative in both eyes. The puppy exhibited slight micropalpebral fissure with both eyes (right eye more than left eye) and mild entropion with both eyes. The cornea, anterior chamber, iris, lens, vitreous and fundus were unremarkable. Dog 6 had a positive direct and consensual pupillary light reflex in both eyes and appeared visual due to successful tracking of a thrown cotton ball. This puppy exhibited severe micropalpebral fissure with both eyes, such that obtaining a Schirmer tear test or tonometry was not possible. Additionally, there was also moderate entropion with both eyes. The eyelid conformation limited the examination of the globe, which appeared within normal limits. The narrowed palpebral fissures and entropion can be seen in Figure 2.

The oral cavities were normal with age-appropriate dentition. Both puppies had large, fluctuant swellings typical of seroma on the caudal aspect of both elbows and tarsi. There were multiple skin lacerations in various stages of healing along with atrophic scars at pressure points on the skin of the elbows and tarsi as well as on the head, neck, pinna of the ears, abdomen, and dorsum in both puppies (Figure 2). The skin was extensible but not necessarily more so than for other puppies of the same breed and age. The carpi and tarsi were moderately hypermobile (Figure 2). Both puppies had medially luxating patellas and crepitus on manipulation of the hips. The remainder of the physical examination was unremarkable.

**Figure 3:**
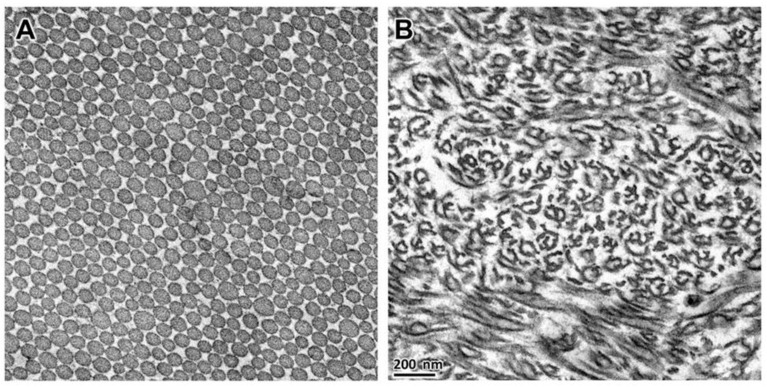
Electron micrographs of dermal-collagen-fibril cross sections from an unaffected 10.5-month-old Dachshund (**A**), and from an affected Alapaha Blue Blood Bulldog puppy (**B**).

**Figure 4:**
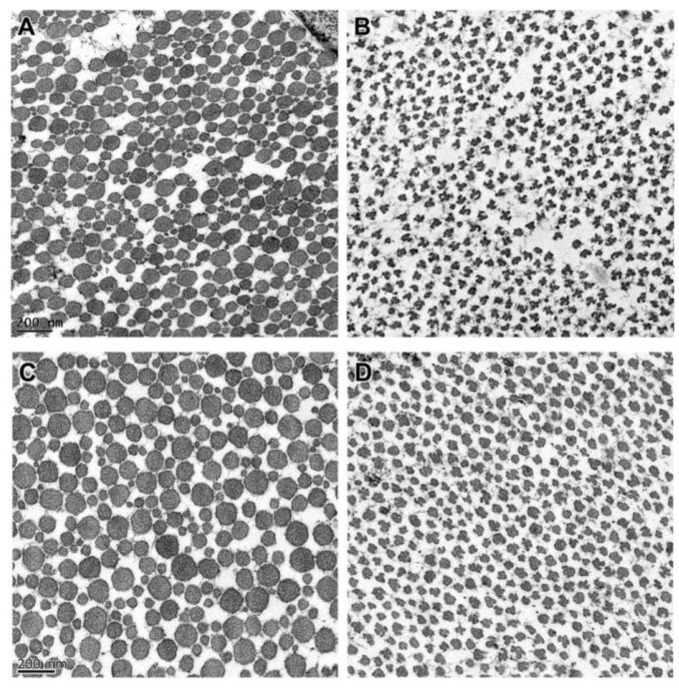
Electron micrographs of collagen-fibril cross sections from the quadriceps tendon of a 10.5-month-old unaffected Dachshund (**A**) and an affected Alapaha Blue Blood Bulldog puppy (**B**). Similar depiction of collagen fibrils from the patellar ligament of the same unaffected Dachshund (**C**), and from an affected Alapaha Blue Blood Bulldog puppy (**D**).

After euthanasia, examination of the connective tissue collagen from skin, tendons and ligaments of Dogs 5 and 6 revealed profound ultrastructural abnormalities. In the dermis of the control dog, the collagen fibrils were circular in cross-section and relatively uniform in size (Figure 3A). In contrast, the dermal collagen fibrils of the affected puppies had curved linear profiles in cross-section (Figure 3B). This ultrastructure has historically been described as hieroglyphic or hieroglyphic-like [23]. The collagen fibrils in tendons and ligaments from an unaffected control dog were circular with variable diameters in cross-section (Figure 4A,C), although the average diameter of the ligament fibrils was greater than that of the tendon. This contrasted with the collagen fibrils in tendons and ligaments from the two affected puppies that were markedly smaller in diameter and irregularly shaped (Figure 4B,D). Compared to the cross-sectional profiles from the affected ligament, the profiles from the tendon were more likely to be smaller and more lobulated. 

**Figure 5:**
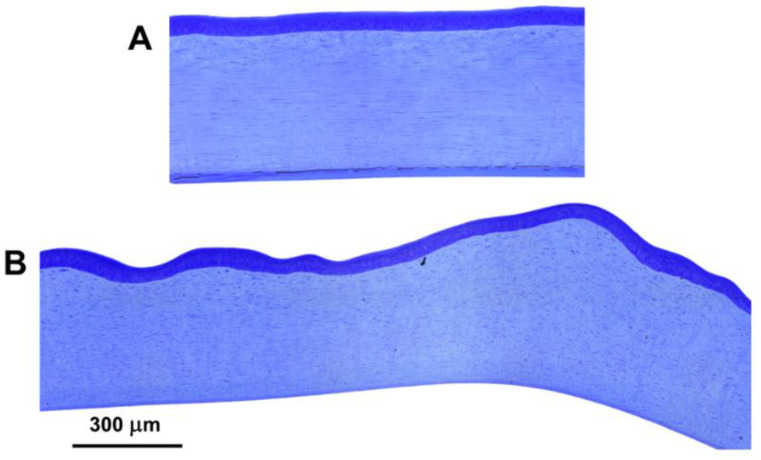
Light micrographs of cross sections of the corneas from an unaffected 10.5-month-old Dachshund (**A**) and from one of the affected Alapaha Blue Blood Bulldog puppies (**B**). Unlike normal corneas, the topography of the front surfaces of the corneas from the affected dogs was quite irregular, although the average corneal thickness was relatively normal. Descemet’s membrane was much thinner in the affected dogs than in the unaffected Dachshund.

**Figure 6:**
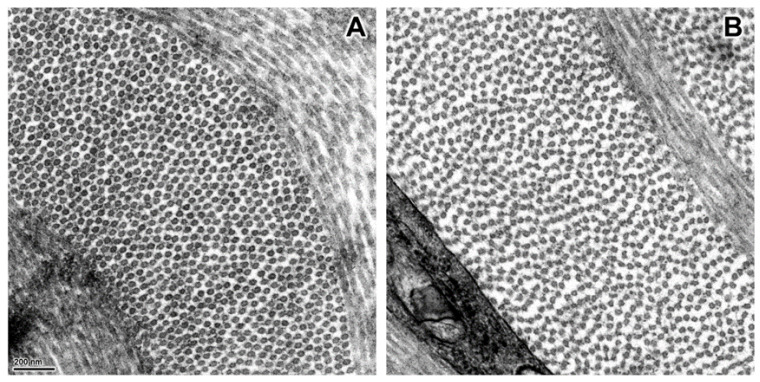
Electron micrographs of cross sections of collagen fibrils from the corneas of an unaffected 10.5-month-old Dachshund (**A**) and from one of the affected Alapaha Blue Blood Bulldog puppies (**B**). In the normal cornea, the collagen fibrils were circular in profile and quite uniform in diameter. The diameters of the corneal collagen fibrils from the affected dogs were similar to those of the normal cornea, but their boundaries were less distinct, and their profiles were more variable. In addition, the packing of the collagen fibrils of the affected dog corneas was looser and less regular than in the cornea from the unaffected dog.

The corneas from Dogs 5 and 6 were irregular in thickness due to variations in the thickness of the stroma (Figure 5). This resulted in a wavy appearance of the epithelial surfaces of the corneas while the inner aspects of the corneas retained normal topography. Descemet’s membrane in these two affected puppies was much thinner than that from the control dog (Figure 5). Accompanying these apparent abnormalities in corneal morphology were subtle differences in corneal collagen ultrastructure between the control and the affected dogs, which had looser packing of the fibrils and more irregularity in fibril cross-sectional profiles (Figure 6).

#### 3.1.4. Dog 7

**Figure 7:**
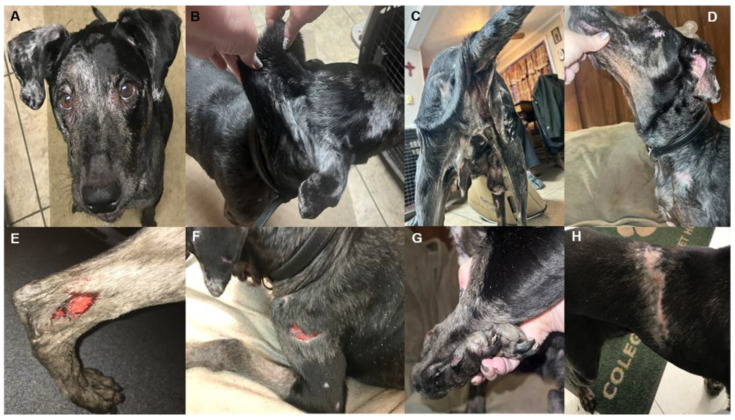
Representative images from a Catahoula Leopard dog illustrating normal palpebral fissures (**A**), hyperextensible skin (**B**), sagging skin (**C**,**D**), typical wounds (**E**,**F**), joint hypermobility (**G**), and atrophic scars (**H**).

We received the clinical history and a blood sample from a 7-year-old, male castrated Catahoula Leopard Dog. Shortly after we acquired the blood sample, the dog was examined by the primary care veterinarian for a routine wellness evaluation, conducted in the owner’s home to avoid skin wounds that might result from transportation in the car. The physical examination revealed hypermobility of the carpal, tarsal, and elbow joints as well as medially luxating patellas (Figure 7). The skin was loose, hyperextensible, and had variably sized atrophic scars that were too numerous to count (Figure 7). The scars were most concentrated on the legs, but distribution was diverse (e.g., head, chest, abdomen, and dorsum of the trunk). The largest scar measured about 7 cm and extended from midline along the caudal dorsal aspect of the back to the right lateral thorax. There were no ocular abnormalities.

The dog had 10 littermates, all of which were unaffected. The sire and dam were clinically unremarkable. When the owner obtained the dog at 8 weeks of age, there were minor wounds on the pinnae that were not identified on any of the littermates. Within 2 days of acquisition, the dog developed several facial wounds that were observed to occur while the dog played with another dog. The owners noticed that the dog would develop wounds with minimal trauma such as walking under the fingernail of the owner or brushing against a shrub. Skin punch biopsies were obtained from normal appearing skin on the trunk when the dog was approximately 1-year-old. According to the pathology report, collagen fibers displayed increased variability in thickness, and often appeared abnormally curled, twisted and hypereosinophilic with prominently increased inter-fiber spaces, especially in the deep dermis. The clinical history combined with the histopathologic abnormalities led to a presumptive diagnosis of EDS.

Over the next 6 years, the dog experienced skin wounds that would heal by second intention approximately once every 2 to 3 months. Skin lacerations that required primary closure by a veterinarian occurred approximately once every 3 months. Wounds healed normally but more severe injuries required more time to resolve. For example, one of the most severe skin lacerations required 8 to 10 weeks to completely heal. The cause for wound development was largely unknown as the dog was always separated from the other dog in the home and kept in a crate when not supervised. Moreover, the dog was always kept attached to a leash while outside. The owner reported three isolated episodes of mild, transient, non-painful swellings around the elbows that resolved without intervention.

At the time of writing, the dog was 9 years of age and had experienced gradual progression in the severity of skin looseness and hyperextensibility. The owner reported that the dog continued to accrue skin wounds from minimal trauma with the same severity and frequency as previously mentioned. The dog developed skin allergies that resulted in a slight increase in frequency of minor wounds that would develop due to self-trauma from licking, scratching, and biting. To mitigate the development of wounds secondary to self-trauma, the owner fitted the dog with clothing that covered most of the body except the head, tail, and distal limbs. The dog was also kept in a padded Elizabethan collar to prevent self-trauma while active wounds healed.

### 3.2. DNA Analysis

The average coverages of the whole genome sequences for Dogs 1, 3 and 7 were 19.5-fold, 21.4-fold, and 46.2-fold, respectively. The tabulated variant reports were filtered for rare, protein-altering variants occurring in the affected dogs, with a focus on variants in orthologs of genes known to be associated with human EDS [2]. Candidate variants were validated by inspection of the aligned reads with IGV. The whole genome sequence from Dog 7 contained a rare homozygous variant, 11:2491238G>A, predicted to produce an altered *ADAMTS2* transcript, c.2897G>A, that encodes a protein in which a conserved argininyl moiety is replaced by a histidyl moiety, p.(Arg966His).

The whole genome sequences from Dogs 1 and 3 each contained the same rare homozygous variant, 11:2280117delC, predicted to produce a frameshift in the transcript from *ADAMTS2*, c.10delC, and encode a severely truncated protein product, p.(Pro4ArgfsTer175). We have used a two-step allelic discrimination assay to genotype 492 individual Alapaha Blue Blood Bulldogs for this deletion. Nine of these dogs including the four described above tested homozygous for the deletion allele, 158 tested heterozygous, and 325 tested homozygous for the reference allele. Thus, the allele frequency was 0.27 for this tested cohort. In addition, we genotyped 217 samples from Pit Bull Terriers in our DNA archives. Dogs 1 and 2 tested homozygous for the deletion allele, all the other Pit Bull Terriers tested homozygous for the reference allele at 11:2280117.

## 4. Discussion

We have identified likely-causal, homozygous *ADAMTS2* variants in 4 of 26 whole genome sequences generated with DNA from dogs that had been diagnosed with EDS. No plausibly causal *ADAMTS2* variants were found in the other 22 whole genome sequences, suggesting that dermatosparaxis is a significant though uncommon subtype of canine EDS. One of the *ADAMTS2* variants discovered by whole genome sequencing has been described in a previous case report [24]. In that case, the likely cause of the EDS in a Doberman Pinscher puppy, hereafter referred to as Dog 8, was a rare, homozygous nonsense mutation, 11:2408978C>T, in the fourth of 22 *ADAMTS2* coding exons. Premature-termination-codon-containing transcripts from that mutant gene were likely depleted by nonsense-mediated decay [41]. Any proteins translated from transcripts that evaded nonsense-mediated decay are predicted to be severely truncated and devoid of enzymatic activity.

In the current report, the whole genome sequences from an EDS-affected Pit Bull Terrier puppy and an EDS-affected Alapaha Blue Blood Bulldog puppy each contained an identical rare, homozygous single-nucleotide deletion and reading-frame shift, 11:2280117delC, in the first coding exon of *ADAMTS2*. Similar to the *ADAMTS2* mutation in Dog 8, this *ADAMTS2* deletion and frameshift is predicted to have produced transcripts for a truncated polypeptide with a premature termination codon rendering them subject to nonsense-mediated decay and, thus, unable to encode a biological active gene product. A third likely-causal *ADAMTS2* variant, 11:2491238G>A, was found in a whole genome sequence that was generated with DNA from an adult Catahoula Leopard Dog with EDS. In this case, the likely cause of the EDS was a rare, homozygous single-nucleotide substitution that produced a nonsynonymous codon change in the 19th *ADAMTS2* coding exon. The mutant gene is predicted to encode a full-length protein product that differs from the normal protein only by the substitution of a histidyl moiety for an argininyl moiety at amino acid 966. Although arginine codons are highly conserved at orthologous positions in mammalian *ADAMTS2* genes, it is plausible, but not demonstrated, that the protein encoded by the Catahoula Leopard Dog variant retained residual procollagen I N-proteinase activity.

As summarized in Table 1, we have compared the molecular-genetic findings and clinical features for eight dogs with dermatosparactic EDS including the four dogs with whole genome sequences. In addition, we included a littermate (Dog 2) of the Pit Bull Terrier with the sequenced genome, a littermate (Dog 4) of the Alapaha Blue Blood Bulldog with the sequenced genome and two Alapaha Blue Blood Bulldog littermates (Dogs 5 and 6) of unknown relation to the Alapaha Blue Blood Bulldog with the sequenced genome. A DNA test confirmed that these four dogs were homozygous for the 11:2280117delC variant described above. Thus, the genomes of all 8 dogs harbored likely-causal *ADAMTS2* alleles in the homozygous state, consistent with reports that dermatosparaxis is a recessive trait in other species [42,43,44].

The most consistent and prominent clinical finding, observed in all eight dogs with the dermatosparactic EDS subtype was fragile skin as evidenced by numerous atrophic scars, lacerations at various stages of healing, and subcutaneous hematomas or seromas. Similar lesions have often been described in previous reports of canine EDS [7,8,9,10,11,12,13,14,15,18,19,21,22,25,26,27,28,29,30,31,32]. Thus, this feature does not distinguish dermatosparactic EDS from other common canine EDS subtypes. For seven of the dogs with dermatosparaxis, fragile skin was the reason that the owners requested their dogs be euthanized. In all seven cases, the euthanasia occurred when the puppies were 8 to 12 weeks old. Dog 8 had a life-threatening degloving injury when euthanized; whereas, the six puppies, with the homozygous 11:2280117delC variant were stable. Although fragile skin is a feature in most canine EDS case reports, we found only two reports that described similar degloving injuries requiring humane euthanasia [11,31]. Another report described EDS-affected littermates that died when four- and eight weeks old, but did not describe the specific circumstances of their death [22]. It is unclear whether Dogs 1 through 6 would have eventually developed life-threatening, degloving injuries or continued to accrue manageable wounds.

The human dermatosparaxis EDS subtype is associated with extreme skin fragility that results in congenital or postnatal skin lacerations, with secondary infections that can lead to premature death [2,34]. Severe wounds continue to develop into adulthood and additional complications can arise such as rectal prolapse and diaphragmatic/umbilical hernia [45]. Extreme skin fragility necessitating euthanasia has also been described in cattle and sheep with the dermatosparaxis subtype of EDS [43,44,46,47]. Nonetheless, less severe forms dermatosparaxis have also been described in human patients and in cattle, sheep and cats [45,48,49,50,51]. The presence or absence of residual procollagen-I-N-proteinase enzymatic activity encoded by the various causal *ADAMTS2* variants most likely influences the inter-species and intra-species differences in clinical severities. For instance, sheep with dermatosparaxis caused by a homozygous nullifying *ADAMTS2* nonsense mutation, typically die or require humane euthanasia in first weeks of life [52,53]. In contrast, sheep with a homozygous *ADAMTS2* missense mutation that encodes a full-length protein with 25% residual procollagen-I-N-proteinase activity have endured frequent lacerations but typically survived to adulthood [48,49].

Like the other dogs with the dermatosparactic EDS subtype, the Catahoula Leopard Dog had fragile skin. In contrast with the dogs that were euthanized as puppies, the Catahoula Leopard Dog has survived to adulthood. The specific reasons for this survival difference are unknown, but likely reflected differences in the criteria that the owners used when considering euthanasia. The four Alapaha Blue Blood Bulldogs were bred to be sold as pets or used in future breeding. Therefore, the identification of a genetic disorder could have influenced the owners’ decisions to euthanize because the dogs could not perform the tasks for which they were bred. The two Pit Bull Terrier puppies were property of an animal shelter, as they had not been adopted. The suspicion of a collagen disorder potentially resulting in lifelong complications likely influenced their decision to euthanize these puppies. In contrast, the Catahoula Leopard Dog was a pet with a dedicated owner that was invested in optimizing the dog’s quality of life. It is also plausible, though not demonstrated, that the *ADAMTS2* variant in the Catahoula Leopard Dog encoded a protein with residual procollagen-I-N-proteinase activity, which decreased clinical severity and has contributed to the continued survival of the dog.

Most of the EDS-affected dogs had joint instability and hyperextensible skin (Table 1). Either or both features have frequently been included in the previously reported clinical histories of dogs with EDS [7,8,9,10,12,14,15,19,22,25,26,27,28,30,54]. Thus, joint instability and hyperextensible skin do not distinguish canine dermatosparaxis from other more common types of canine EDS.

Most of the Alapaha Blue Blood Bulldogs and Pit Bull Terrier puppies in this study had swellings associated with one or more of their elbows, tarsi, carpi, or paws. These swellings were often painful, bruised, exuded discharge, and had wounds at varying stages of healing. A similar description of painful swelling around the carpi and tarsi was reported in Dog 8 [24]. Swellings around appendicular joints have been reported in at least three previous publications about canine EDS [7,28,54]. Another case report described occasional hematomas on the legs, but did not indicate whether or not the hematomas were associated with joints [22]. Nonetheless, descriptions of swellings around appendicular joints are not common in previous clinical descriptions of canine EDS, suggesting that this sign may be indicative of severe canine dermatosparaxis. Appendicular joint swellings were infrequent occurrences in the long clinical history of the affected Catahoula Leopard Dog, consistent with the possibility that this dog may have a less severe form of dermatosparaxis. The specific causes for appendicular joint swellings are unknown and likely complex. One possibility is that these regions are at pressure points that are frequently exposed to varying magnitudes of injury and subsequent seroma formation, infection, or both. Another potential explanation for the frequent appendicular joint-associated swellings is that they resulted from, or were exacerbated by, joint instability; however, the joint-swellings in the EDS-affected Pit Bull Terrier and Alapaha Blue Blood Bulldog puppies were more severe than would be expected from joint instability alone.

Bilateral, narrowed palpebral fissures were a distinct clinical feature in five of the six dermatosporactic Pit Bull Terrier and Alapaha Blue Blood Bulldog puppies (Table 1). This feature has been used by experienced breeders of Alapaha Blue Blood Bulldogs for early identification of affected puppies (personal communication: G.S.J.). Narrowed palpebral fissures were not mentioned in the clinical descriptions of the Dogs 7 and 8; however, Dog 8 had ocular chemosis or conjunctival edema. We have not found mention of micropalpebral fissures in previous reports about canine EDS. Nonetheless, infants with dermatosparaxis typically have swollen, edematous eyelids and excessive periorbital skin [42,45,55,56]. Edema of the eyelids and other areas of the skin has been reported in ruminates with dermatosparaxis [43,57,58]. In affected human infants, the severity of the periocular edema slowly diminishes with age [55]. If the edema resolves with age in dogs with dermatosparaxis, it is plausible that Dog 7 had unrecognized or forgotten periocular edema as a young puppy. There is no obvious cause-and-effect relationship between defective collagen fibrillogenesis and lymphedema. In this regard, it may be pertinent that, besides the fibrillar procollagens, a variety of unrelated proteins are efficiently cleaved by the procollagen-aminopeptidases encoded by *ADAMTS2*, *ADAMTS3*, and *ADAMTS14* [35]. One such protein is vascular endothelial growth factor C (VEGF-C) which plays an essential role in embryonic lymphangiogenesis and may be required for normal growth and development of the lymphatic system [59,60,61]. VEGF-C is secreted in an inactive form that requires proteolytic removal of an amino-terminal propeptide for activation. In the embryo, VEGF-C is activated by the peptidase encoded by *ADAMTS3* [62]; however, based on developmental and spatial expression patterns, it is likely that the procollagen-aminopeptidase encoded by *ADAMTS2* activates VEGF-C in the dermis during perinatal development and beyond [61]. Periocular edema may be a distinguishing feature in young puppies with dermatosparaxis and narrowed palpebral fissures may be a breed-specific or breed-type-specific expression of that feature.

Intermittent ataxia was observed in one of the affected Pit Bull Terrier puppies (Dog 1) and in one of the affected Alapaha Blue Blood Bulldogs (Dog 4). These observations were unexpected, because we found no mention of ataxia or episodic ataxia in the clinical histories of humans or animals with EDS attributed to *ADAMTS2* variants. However, delayed gross motor development has been reported in some children with the dermatosparaxis EDS subtype [45]. Complete neurological examinations were not performed in the two puppies with suspected ataxia. Thus, this observation must be interpreted cautiously. It is possible that suboptimal coordination expected in a young developing puppy combined with joint swelling and pain could have been mistaken for ataxia. Potential neurological involvement of the dermatosparaxis subtype of EDS in dogs warrants future investigation.

The regularly spaced circular profiles of collagen fibrils in the electron micrograph of the control-dog dermis (Figure 3A) are typical of the normal cross-sectional ultrastructure of canine dermal collagen fibrils [63], but distinct from the ultrastructure of collagen fibril cross sections from the dermis of Dogs 5 and 6. Similar abnormal collagen fibril cross-sectional ultrastructure, which has been described by others as hieroglyphic-like, have been identified in dermal samples from human, bovine, ovine and canine EDS patients [23,46,57,64]. This abnormal ultrastructure has been considered pathognomonic for severe dermatosparaxis [42] and diagnoses have been based partly or completely on this laboratory finding [23,65]. Nonetheless, less extreme deviations from the normal ultrastructure have been found in the dermis of individuals with less severe forms of dermatosparaxis [45,49,51,66], and also in dermal samples from human patients with the Arthrochalasia subtype of EDS, characterized by articular hypermobility, dislocations and subluxations, and by fragile tissue and hyperextensible skin [67]. Arthrochalasia results from heterozygous *COL1A1* or *COL1A2* mutations that disrupt the procollagen-aminopeptidase cleavage sites in the encoded proteins resulting in the retention of the aminopropeptide on some of the expressed α1(I) or α2(I) subunits [2,34,67,68].

A plausible explanation for the abnormal cross-sectional ultrastructure of dermal collagen fibrils from individuals with dermatosparaxis or arthrochalasia is that tropocollagen molecules with retained aminopropeptides are incorporated into fibrils during fibrillogenesis, but constrained to the collagen-fibril surface [69,70,71]. At some point, increasing the fraction of tropocollagen molecules with retained aminopropeptides will saturate available sites on the cylindrical fibril surface forcing microanatomical adjustments that increase the surface-to-mass ratios. This can be accomplished by decreasing the cross-sectional diameters of the cylindrical fibrils and/or by altering the normally round cross-sectional profiles. Minor ultrastructural changes may appear as serrated circles such as those from patients with arthrochalasia caused by *COL1A2* mutations [72] or mild dermatosparaxis [45,46]. Compact “hieroglyphs” have been reported from dermal tissue from patients with arthrochalasia caused by *COL1A1* mutations [67] and moderately severe cases of dermatosparaxis [51,73]. Because a high proportion of the procollagen-aminopeptidase activity expressed in the dermis of post-natal mammals is encoded by *ADAMTS2* [61], a high proportion of dermal fibrils in individuals with dermatosparaxis due to homozygous nullifying *ADAMTS2* mutations have retained aminopropeptides. The fibrils composed of these modified tropocollagen molecules assume a ribbon- or sheet-like confirmation [69], which may correspond to the curved linear figures seen in fibril cross sections from Dogs 5 and 6 (Figure 3B).

At least seven earlier canine-EDS case reports or reviews have included transmission electron microscopy images showing dermal-collagen fibril ultrastructure [13,14,22,23,26,27,30]; however, only the image provided by Holbrook and Byers with no accompanying clinical information [23], resembles the dermal collagen-fibril cross-sectional ultrastructures from Dogs 5 and 6 (Figure 3B). Most of the other images in the previous reports were difficult for us to interpret. Nonetheless, the fibril cross sections from an EDS-affected Garafiano Shepherd appeared as compact hieroglyphs [22], perhaps resulting from a less severe case of dermatosparaxis or from arthrochalasia. The image from an EDS-affected Greyhound showed mostly normal circular cross sections with occasional cauliflower formations [14], typical of the human “Classical” EDS subtype [74]. Finally, the image from a cross-bred dog showed circular cross sections with a wide range of diameters [30], typical of the human “Vascular” EDS subtype [74] and consistent with the report of the cross-bred dog’s sudden death from a subclavian artery rupture.

The abnormalities in the cross-sectional ultrastructure of collagen fibrils from the tendons and ligaments of Dogs 5 and 6 were less extreme than those from the dermis and resembled the cross-sectional ultrastructure of fibrils from the dermis of individuals with less severe forms of dermatosparaxis and from patients with arthrochalasia. Similarly, the abnormalities in the cross-sectional ultrastructures of collagen fibrils from the tendons of dermatosparactic calves and lambs were less extreme than those from the dermis [33,35,57]. Furthermore, in cattle the proportion of type I collagen subunits with retained aminopropeptides is higher in the dermis than in tendons [43]. A plausible explanation for these tissue-specific differences is that nearly all of the procollagen-aminopeptidase activity in the dermis is encoded by *ADAMTS2*; whereas, in the other tissues additional procollagen-aminopeptidases such as those encoded by *ADAMTS3* and *ADAMTS14* are able to partially compensate for the *ADAMTS2* deficiency [75].

The cornea and sclera comprise a protective outer surface of the eye. This requires tensile strength, which is largely provided by collagen fibrils, as it is in the dermis and in tendons and ligaments. The corneal collagen fibrils are the relatively narrow and distributed within interlacing lamellae in which the fibrils are aligned in parallel [76,77]. Unlike the sclera, dermis, tendons and ligaments, the normal cornea must be transparent and refract visible light. Transparency in the cornea is thought to occur because within the lamellae, light scatter from individual fibrils interferes with light scatter from neighboring fibrils such that they cancel out in all directions except the forward direction [76]. Although the precise structural constraints for corneal transparency have not been worked out, it appears that important contributions are made by the narrowness of the corneal fibrils and by the uniformity of both the cross-sectional diameters and the intra-fibril distances [76,78]. The transparency of corneas from individuals with the dermatosparaxis and the minimal changes to corneal fibril ultrastructure in the affected Alapaha Blue Blood Bulldogs (Figure 6) suggest that *ADAMTS2* has a relatively minor role in collagen fibrillogenesis in the cornea.

Without establishment of the normal range in age-matched control dogs, the significance of the marked thickness difference in Descemet’s membrane between affected and control samples (Figure 7) remains uncertain. The post-natal thickness of the Descemet’s membrane increases continuously with age [79]. The affected Alapaha Blue Blood Bulldogs puppies were 8 weeks old, whereas the control Dachshund was 10.5 months old. It is plausible that this age difference is responsible for some or all of the thickness difference. The wavy corneal surface shown in Figure 7 was unexpected and warrants further investigation. It may reflect reduced rigidity of the cornea that only became apparent after dissection of the cornea from the eye.

We have received samples from 167 Alapaha Blue Blood Bulldogs that harbored at least one likely-causal 11:2280117delC allele. The calculated variant-allele frequency of 0.27 for the cohort of genotyped Alapaha Blue Blood Bulldogs might not accurately estimate the frequency of the variant allele among the general population of Alapaha Blue Blood Bulldogs. Nonetheless, there are no previous reports of wide-spread distributions of EDS-causing alleles among privately owned dogs. The accumulation of the likely-causal alleles among Alapaha Blue Blood Bulldogs may have resulted from the extensive influence of one or a few heterozygous popular sires in this relatively rare dog breed. Alternatively, careful examination of phenotypic features shared by heterozygous dogs in comparison to those from dogs homozygous for the *ADAMTS2* reference allele may reveal a basis for selection of heterozygotes by Alapaha Blue Blood Bulldog breeders.

In contrast to the situation with Alapaha Blue Blood Bulldogs, the likely-causal 11:2280117delC allele was rare among genotyped Pit Bull Terriers. Pit Bull Terriers and Alapaha Blue Blood Bulldogs are similar in appearance, although members of the latter-mentioned breed tend to be larger. It is plausible that shelter workers were mistaken when they concluded that Dogs 1 and 2 and their relatives were Pit Bull Terriers and that these Dogs 1 and 2 were, in fact, Alapaha Blue Blood Bulldogs. In any event, genotyping Pit Bull Terriers for the deletion at 11:2280117 is unlikely to be cost effective unless the subject dogs or their close relatives have shown clinical signs of EDS.

As discussed in the introduction, human EDS cases are currently subclassified, based on medical histories, laboratory findings, patterns of inheritance, and the identities of the genes that harbor the causal variants [2]. Hesitancy to switch to a less complex system based solely on the identity of the causal gene may have, in part, resulted from a reluctance to abandon the current system because it has been broadly accepted and used in numerous case reports and scientific investigations and because it provides a framework for extensive, standardized clinical workups. Neither of these reasons apply to canine EDS, where few previous case reports have classified the disease beyond a general diagnosis of EDS and where minimal clinical workups are the norm. To promote a practical, causal-gene based system for the subclassification of canine EDS, we encourage clinicians and investigators with novel cases of canine EDS to establish the molecular-genetic causes by contacting one of the several research laboratories around the world that routinely use whole-genome sequencing to establish the likely causes for heritable canine diseases, often at little or no cost to the dog owner.

## 5. Conclusions

This report has expanded the genotypic and phenotypic spectrum of the dermatosparaxis subtype of canine EDS by identifying two novel *ADAMTS2* variants that, in the homozygous state, were the likely causes for the dermatosparactic form of canine EDS and by documenting the associated clinical histories and laboratory findings for seven dogs with EDS likely caused by these variants. The clinical histories for these seven dogs were discussed in combination with the previously described clinical history of a Doberman Pinscher puppy with EDS likely caused by a homozygous *ADAMTS2* nonsense mutation. These clinical histories and associated postmortem findings were compared to those in earlier descriptions of canine EDS and to descriptions of dermatosparaxis in other species.

We believe this report demonstrates the potential utility of subclassifying canine EDS according to the identity of the gene harboring the likely-causal genetic variant. The accurate diagnosis of canine EDS subtypes should facilitate a precision-medicine based approach for optimizing prognosis and management in future cases of canine EDS.

## Figures and Tables

**Table 1:** Molecular-genetic and clinical features of dogs with dermatosparactic Ehlers-Danlos Syndrome.

Dog Identity	Dog 1	Dog 2	Dog 3	Dog 4	Dog 5	Dog 6	Dog 7	Dog 8
reed	Pit Bull Terrier	Pit Bull Terrier	Alapaha Blue Blood Bulldog	Alapaha Blue Blood Bulldog	Alapaha Blue Blood Bulldog	Alapaha Blue Blood Bulldog	Catahoula Leopard Dog	Doberman Pinscher
Mutation	11:2280117delC	11:2280117delC	11:2280117delC	11:2280117delC	11:2280117delC	11:2280117delC	11:2491238G>A	11:2408978C>T
Mutation type	Frameshift	Frameshift	Frameshift	Frameshift	Frameshift	Frameshift	Missense	Nonsense
Mutation zygosity	Homozygous	Homozygous	Homozygous	Homozygous	Homozygous	Homozygous	Homozygous	Homozygous
Euthanasia	Yes	Yes	Yes	Yes	Yes	Yes	No	Yes
Age at death	8 Weeks	8 Weeks	12 Weeks	12 Weeks	8 Weeks	8 Weeks	>9 Years	8 Weeks
Fragile skin	Yes	Yes	Yes	Yes	Yes	Yes	Yes	Yes
Atrophic scars	Yes	Yes	Yes	Yes	Yes	Yes	Yes	Yes
Hyper-extensible Skin	Yes	Yes	Yes	Yes	No	No	Yes	Yes
Joint instability	Yes	No	Yes	Yes	Yes	Yes	Yes	Yes
Swollen joints	Yes	No	Yes	Yes	Yes	Yes	Infrequent	Yes
Periocular lesions	Micropalpebral fissures	None	Micropalpebral fissures	Micropalpebral fissures	Micropalpebral fissures	Micropalpebral fissures	None	Ocular Chemosis
Ataxia	Yes	No	No	Yes	No	No	No	No

## Data Availability

The whole-genome-sequence data used in this study are available in the NCBI SRA (https://trace.ncbi.nlm.nih.gov/Traces/sra/sra.cgi, accessed on 15 March 2022). The SRA accession numbers are provided in the Supplemental Table S1.

## Supplementary Materials

The following supporting information can be downloaded at: https://www.mdpi.com/article/10.3390/genes13112158/s1, Table S1: SRA BioSample IDs and metadata for dogs sequenced in this study and control samples used in the WGS analysis cohort.

## References

[1] Bloom L., Byers P., Francomano C., Tinkle B., Malfait F., Steering Committee of the International Consortium on the Ehlers-Danlos Syndromes (2017). The international consortium on the Ehlers-Danlos syndromes. Am. J. Med. Genet. C Semin. Med. Genet..

[2] Malfait F., Castori M., Francomano C.A., Giunta C., Kosho T., Byers P.H. (2020). The Ehlers-Danlos syndromes. Nat. Rev. Dis. Prim..

[3] Byers P.H., Belmont J., Black J., De Backer J., Frank M., Jeunemaitre X., Johnson D., Pepin M., Robert L., Sanders L. (2017). Diagnosis, natural history, and management in vascular Ehlers-Danlos syndrome. Am. J. Med. Genet. C Semin. Med. Genet..

[4] Cortini F., Villa C., Marinelli B., Combi R., Pesatori A.C., Bassotti A. (2019). Understanding the basis of Ehlers-Danlos syndrome in the era of the next-generation sequencing. Arch. Dermatol. Res..

[5] Desai A., Connolly J.J., March M., Hou C., Chiavacci R., Kim C., Lyon G., Hadley D., Hakonarson H. (2016). Systematic data-querying of large pediatric biorepository identifies novel Ehlers-Danlos Syndrome variant. BMC Musculoskelet. Disord..

[6] Steinle J., Hossain W.A., Lovell S., Veatch O.J., Butler M.G. (2021). ADAMTSL2 gene variant in patients with features of autosomal dominant connective tissue disorders. Am. J. Med. Genet. A.

[7] Bauer A., Bateman J.F., Lamande S.R., Hanssen E., Kirejczyk S.G.M., Yee M., Ramiche A., Jagannathan V., Welle M., Leeb T. (2019). Identification of Two Independent COL5A1 Variants in Dogs with Ehlers-Danlos Syndrome. Genes.

[8] Bauer A., de Lucia M., Leuthard F., Jagannathan V., Leeb T. (2019). Compound heterozygosity for TNXB genetic variants in a mixed-breed dog with Ehlers-Danlos syndrome. Anim. Genet..

[9] Kiener S., Chevallier L., Jagannathan V., Briand A., Cochet-Faivre N., Reyes-Gomez E., Leeb T. (2022). A COL5A2 In-Frame Deletion in a Chihuahua with Ehlers-Danlos Syndrome. Genes.

[10] Anderson J.H., Brown R.E. (1978). Cutaneous asthenia in a dog. J. Am. Vet. Med. Assoc..

[11] Arlein M.S. (1947). Generalized acute cutaneous asthenia in a dog. J. Am. Vet. Med. Assoc..

[12] Barnett K., Cottrell B.D. (1987). Ehlers-Danlos syndrome in a dog: Ocular, cutaneous and articular abnormalities. J. Small Anim. Pract..

[13] Bellini M.H., Caldini E.T., Scapinelli M.P., Simoes M.J., Machado D.B., Nurmberg R. (2009). Increased elastic microfibrilsand thickening of fibroblastic nuclear lamina in canine cutaneous asthenia. Vet. Dermatol..

[14] Cahill J.I., Jones B.R., Barnes G.R., Craig A.S. (1980). A collagen dysplasia in a greyhound bitch. N. Z. Vet. J..

[15] Freeman L.J., Hegreberg G.A., Robinette J.D. (1987). Ehlers-Danlos syndrome in dogs and cats. Semin. Vet. Med. Surg..

[16] Freeman L.J., Hegreberg G.A., Robinette J.D. (1989). Cutaneous wound healing in Ehlers-Danlos syndrome. Vet. Surg..

[17] Freeman L.J., Hegreberg G.A., Robinette J.D., Kimbrell J.T. (1989). Biomechanical properties of skin and wounds in Ehlers-Danlos syndrome. Vet. Surg..

[18] Gething M.A. (1971). Suspected Ehlers-Danlos syndrome in the dog. Vet. Rec..

[19] Hegreberg G.A. (1975). Animal model of human disease: Ehlers-Danlos syndrome. Am. J. Pathol..

[20] Hegreberg G.A., Padgett G.A., Henson J.B. (1970). Connective tissue disease of dogs and mink resembling Ehlers-Danlos syndrome of man. 3. Histopathologic changes of the skin. Arch. Pathol..

[21] Hegreberg G.A., Padgett G.A., Ott R.L., Henson J.B. (1970). A heritable connective tissue disease of dogs and mink resembling Ehlers-Danlos syndrome of man. I. Skin tensile strength properties. J. Investig. Dermatol..

[22] Rodriguez F., Herraez P., de los Monteros A.E., Calabuig P., Rodriguez J.L. (1996). Collagen dysplasia in a litter of Garafiano shepherd dogs. Zent. Vet. A.

[23] Holbrook K.A., Byers P.H. (1982). Structural abnormalities in the dermal collagen and elastic matrix from the skin of patients with inherited connective tissue disorders. J. Investig. Dermatol..

[24] Jaffey J.A., Bullock G., Teplin E., Guo J., Villani N.A., Mhlanga-Mutangadura T., Schnabel R.D., Cohn L.A., Johnson G.S. (2019). A homozygous ADAMTS2 nonsense mutation in a Doberman Pinscher dog with Ehlers Danlos syndrome and extreme skin fragility. Anim. Genet..

[25] Matthews B.R., Lewis G.T. (1990). Ehlers-Danlos syndrome in a dog. Can. Vet. J..

[26] Minor R.R., Lein D.H., Patterson D.F., Krook L., Porter T.G., Kane A.C. (1983). Defects in collagen fibrillogenesis causing hyperextensible, fragile skin in dogs. J. Am. Vet. Med. Assoc..

[27] Paciello O., Lamagna F., Lamagna B., Papparella S. (2003). Ehlers-Danlos-like syndrome in 2 dogs: Clinical, histologic, and ultrastructural findings. Vet. Clin. Pathol..

[28] Poulsen P.H., Thomsen M.K., Kristensen F. (1985). Cutaneous asthenia in the dog. A report of two cases. Nord. Vet. Med..

[29] Rasch S.N. (2017). Surgical and medical treatment of ocular disease in a dog with Ehlers-Danlos syndrome. Clin. Case Rep..

[30] Uri M., Verin R., Ressel L., Buckley L., McEwan N. (2015). Ehlers-Danlos syndrome associated with fatal spontaneous vascular rupture in a dog. J. Comp. Pathol..

[31] Wall R.D. (1947). Congenital defect of the skin. N. Am. Vet..

[32] Ward G.W. (1970). Cutaneous asthenia (*cutis hyperelastica*) of dogs. Aust. Vet. J..

[33] Colige A., Sieron A.L., Li S.W., Schwarze U., Petty E., Wertelecki W., Wilcox W., Krakow D., Cohn D.H., Reardon W. (1999). Human Ehlers-Danlos syndrome type VII C and bovine dermatosparaxis are caused by mutations in the procollagen I N-proteinase gene. Am. J. Hum. Genet..

[34] Brady A.F., Demirdas S., Fournel-Gigleux S., Ghali N., Giunta C., Kapferer-Seebacher I., Kosho T., Mendoza-Londono R., Pope M.F., Rohrbach M. (2017). The Ehlers-Danlos syndromes, rare types. Am. J. Med. Genet. C Semin. Med. Genet..

[35] Bekhouche M., Leduc C., Dupont L., Janssen L., Delolme F., Vadon-Le Goff S., Smargiasso N., Baiwir D., Mazzucchelli G., Zanella-Cleon I. (2016). Determination of the substrate repertoire of ADAMTS2, 3, and 14 significantly broadens their functions and identifies extracellular matrix organization and TGF-beta signaling as primary targets. FASEB J..

[36] Roberts J.H., Halper J. (2021). Connective Tissue Disorders in Domestic Animals. Adv. Exp. Med. Biol..

[37] Katz M.L., Khan S., Awano T., Shahid S.A., Siakotos A.N., Johnson G.S. (2005). A mutation in the CLN8 gene in English Setter dogs with neuronal ceroid-lipofuscinosis. Biochem. Biophys. Res. Commun..

[38] Zeng R., Coates J.R., Johnson G.C., Hansen L., Awano T., Kolicheski A., Ivansson E., Perloski M., Lindblad-Toh K., O’Brien D.P. (2014). Breed distribution of SOD1 alleles previously associated with canine degenerative myelopathy. J. Vet. Intern. Med..

[39] Hoeppner M.P., Lundquist A., Pirun M., Meadows J.R., Zamani N., Johnson J., Sundstrom G., Cook A., FitzGerald M.G., Swofford R. (2014). An improved canine genome and a comprehensive catalogue of coding genes and non-coding transcripts. PLoS ONE.

[40] Mowjoodi A., Paton T.A., Scherer S.W. (2014). Discrimination of SNPs in GC-rich regions using a modified hydrolysis probe chemistry protocol. Biotechniques.

[41] Lindeboom R.G., Supek F., Lehner B. (2016). The rules and impact of nonsense-mediated mRNA decay in human cancers. Nat. Genet..

[42] Malfait F., De Coster P., Hausser I., van Essen A.J., Franck P., Colige A., Nusgens B., Martens L., De Paepe A. (2004). The natural history, including orofacial features of three patients with Ehlers-Danlos syndrome, dermatosparaxis type (EDS type VIIC). Am. J. Med. Genet. A.

[43] Hanset R., Lapiere C.M. (1974). Inheritance of dermatosparaxis in the calf. A genetic defect of connective tissues. J. Hered..

[44] Helle O., Nes N.N. (1972). A hereditary skin defect in sheep. Acta Vet. Scand..

[45] Van Damme T., Colige A., Syx D., Giunta C., Lindert U., Rohrbach M., Aryani O., Alanay Y., Simsek-Kiper P.O., Kroes H.Y. (2016). Expanding the clinical and mutational spectrum of the Ehlers-Danlos syndrome, dermatosparaxis type. Genet. Med..

[46] O’Hara P.J., Read W.K., Romane W.M., Bridges C.H. (1970). A collagenous tissue dysplasia of calves. Lab. Investig..

[47] Hanset R., Ansay M. (1967). Dermatosparaxie (peau déchirée) chez le veau: Un défaut général du tissu conjonctif, de nature héréditaire. Ann. Med. Vet..

[48] Ramshaw J.A. (1984). A mild form of ovine dermatosparaxis. Collagen Relat. Res..

[49] Bavinton J.H., Peters D.E., Ramshaw J.A. (1985). A morphologic study of a mild form of ovine dermatosparaxis. J. Investig. Dermatol..

[50] Counts D.F., Byers P.H., Holbrook K.A., Hegreberg G.A. (1980). Dermatosparaxis in a Himalayan cat: I. Biochemical studies of dermal collagen. J. Investig. Dermatol..

[51] Holbrook K.A., Byers P.H., Counts D.F., Hegreberg G.A. (1980). Dermatosparaxis in a Himalayan cat: II. Ultrastructural studies of dermal collagen. J. Investig. Dermatol..

[52] Zhou H., Hickford J.G., Fang Q. (2012). A premature stop codon in the ADAMTS2 gene is likely to be responsible for dermatosparaxis in Dorper sheep. Anim. Genet..

[53] Vaatstra B., Halliday W., Waropastrakul S. (2011). Dermatosparaxis in two White Dorper lambs. N. Z. Vet. J..

[54] Keep J.M. (1969). Cutis hyperelastica in a dog. Aust. Vet. J..

[55] Wertelecki W., Smith L.T., Byers P. (1992). Initial observations of human dermatosparaxis: Ehlers-Danlos syndrome type VIIC. J. Pediatr..

[56] Fujimoto A., Wilcox W.R., Cohn D.H. (1997). Clinical, morphological, and biochemical phenotype of a new case of Ehlers-Danlos syndrome type VIIC. Am. J. Med. Genet..

[57] Fjolstad M., Helle O. (1974). A hereditary dysplasia of collagen tissues in sheep. J. Pathol..

[58] Atroshi F., Henriksson K., Lindberg L.A., Multia M. (1983). A heritable disorder of collagen tissue in Finnish crossbred sheep. Zent. Vet. A.

[59] Jha S.K., Rauniyar K., Jeltsch M. (2018). Key molecules in lymphatic development, function, and identification. Ann. Anat..

[60] Jeltsch M., Jha S.K., Tvorogov D., Anisimov A., Leppanen V.M., Holopainen T., Kivela R., Ortega S., Karpanen T., Alitalo K. (2014). CCBE1 enhances lymphangiogenesis via A disintegrin and metalloprotease with thrombospondin motifs-3-mediated vascular endothelial growth factor-C activation. Circulation.

[61] Dupont L., Joannes L., Morfoisse F., Blacher S., Monseur C., Deroanne C.F., Noel A., Colige A.C. (2022). ADAMTS2 and ADAMTS14 can substitute for ADAMTS3 in adults for pro-VEGFC activation and lymphatic homeostasis. JCI Insight.

[62] Janssen L., Dupont L., Bekhouche M., Noel A., Leduc C., Voz M., Peers B., Cataldo D., Apte S.S., Dubail J. (2016). ADAMTS3 activity is mandatory for embryonic lymphangiogenesis and regulates placental angiogenesis. Angiogenesis.

[63] Ueda K., Kawai T., Senoo H., Shimizu A., Ishiko A., Nagata M. (2018). Histopathological and electron microscopic study in dogs with patellar luxation and skin hyperextensibility. J. Vet. Med. Sci..

[64] Nusgens B.V., Verellen-Dumoulin C., Hermanns-Le T., De Paepe A., Nuytinck L., Pierard G.E., Lapiere C.M. (1992). Evidence for a relationship between Ehlers-Danlos type VII C in humans and bovine dermatosparaxis. Nat. Genet..

[65] Lapiere C.M., Nusgens B.V. (1993). Ehlers-Danlos type VII-C, or human dermatosparaxis. The offspring of a union between basic and clinical research. Arch. Dermatol..

[66] Holm D.E., van Wilpe E., Harper C.K., Duncan N.M. (2008). The occurrence of dermatosparaxis in a commercial Drakensberger cattle herd in South Africa. J. S. Afr. Vet. Assoc..

[67] Giunta C., Chambaz C., Pedemonte M., Scapolan S., Steinmann B. (2008). The arthrochalasia type of Ehlers-Danlos syndrome (EDS VIIA and VIIB): The diagnostic value of collagen fibril ultrastructure. Am. J. Med. Genet. A.

[68] Byers P.H., Duvic M., Atkinson M., Robinow M., Smith L.T., Krane S.M., Greally M.T., Ludman M., Matalon R., Pauker S. (1997). Ehlers-Danlos syndrome type VIIA and VIIB result from splice-junction mutations or genomic deletions that involve exon 6 in the COL1A1 and COL1A2 genes of type I collagen. Am. J. Med. Genet..

[69] Hulmes D.J., Kadler K.E., Mould A.P., Hojima Y., Holmes D.F., Cummings C., Chapman J.A., Prockop D.J. (1989). Pleomorphism in type I collagen fibrils produced by persistence of the procollagen N-propeptide. J. Mol. Biol..

[70] Watson R.B., Wallis G.A., Holmes D.F., Viljoen D., Byers P.H., Kadler K.E. (1992). Ehlers Danlos syndrome type VIIB. Incomplete cleavage of abnormal type I procollagen by *N*-proteinase in vitro results in the formation of copolymers of collagen and partially cleaved pNcollagen that are near circular in cross-section. J. Biol. Chem..

[71] Watson R.B., Holmes D.F., Graham H.K., Nusgens B.V., Kadler K.E. (1998). Surface located procollagen *N*-propeptides on dermatosparactic collagen fibrils are not cleaved by procollagen *N*-proteinase and do not inhibit binding of decorin to the fibril surface. J. Mol. Biol..

[72] Eyre D.R., Shapiro F.D., Aldridge J.F. (1985). A heterozygous collagen defect in a variant of the Ehlers-Danlos syndrome type VII. Evidence for a deleted amino-telopeptide domain in the pro-alpha 2(I) chain. J. Biol. Chem..

[73] Colige A., Nuytinck L., Hausser I., van Essen A.J., Thiry M., Herens C., Ades L.C., Malfait F., Paepe A.D., Franck P. (2004). Novel types of mutation responsible for the dermatosparactic type of Ehlers-Danlos syndrome (Type VIIC) and common polymorphisms in the ADAMTS2 gene. J. Investig. Dermatol..

[74] Sobey G. (2015). Ehlers-Danlos syndrome: How to diagnose and when to perform genetic tests. Arch. Dis. Child..

[75] Le Goff C., Somerville R.P., Kesteloot F., Powell K., Birk D.E., Colige A.C., Apte S.S. (2006). Regulation of procollagen amino-propeptide processing during mouse embryogenesis by specialization of homologous ADAMTS proteases: Insights on collagen biosynthesis and dermatosparaxis. Development.

[76] Meek K.M., Knupp C. (2015). Corneal structure and transparency. Prog. Retin. Eye Res..

[77] Espana E.M., Birk D.E. (2020). Composition, structure and function of the corneal stroma. Exp. Eye Res..

[78] Meek K.M., Leonard D.W., Connon C.J., Dennis S., Khan S. (2003). Transparency, swelling and scarring in the corneal stroma. Eye.

[79] de Oliveira R.C., Wilson S.E. (2020). Descemet’s membrane development, structure, function and regeneration. Exp. Eye Res..

